# Local chronicles reveal the effect of anthropogenic and climatic impacts on local extinctions of Chinese pangolins (*Manis pentadactyla*) in mainland China

**DOI:** 10.1002/ece3.9388

**Published:** 2022-10-05

**Authors:** Haiyang Gao, Hongliang Dou, Shichao Wei, Song Sun, Yulin Zhang, Yan Hua

**Affiliations:** ^1^ Guangdong Provincial Key Laboratory of Silviculture, Protection and Utilization Guangdong Academy of Forestry Guangzhou China

**Keywords:** climate change, extinction risk assessment, historical data, human interference, pangolin conservation

## Abstract

Anthropogenic and climatic factors affect the survival of animal species. Chinese pangolin is a critically endangered species, and identifying which variables lead to local extinction events is essential for conservation management. Local chronicles in China serve as long‐term monitoring data, providing a perspective to disentangle the roles of human impacts and climate changes in local extinctions. Therefore, we established generalized additive models to identify factors leading to local extinction with historical data from 1700–2000 AD in mainland China. Then we decreased the time scale and constructed extinction risk models using MaxEnt in a 30‐year transect (1970–2000 AD) to further assess extinction probability of extant Chinese pangolin populations. Lastly, we used principal component analysis to assess variation of related anthropogenic and climatic variables. Our results showed that the extinction probability increased with global warming and human population growth. An extinction risk assessment indicated that the population and distribution range of Chinese pangolins had been persistently shrinking in response to highly intensive human activities (main cause) and climate change. PCA results indicated that variability of climatic variables is greater than anthropogenic variables. Overall, the factors causing local extinctions are intensive human interference and drastic climatic fluctuations which induced by the effect of global warming. Approximately 28.10% of extant Chinese pangolins populations are confronted with a notable extinction risk (0.37 ≤ extinction probability≤0.93), specifically those in Southeast China, including Guangdong, Jiangxi, Zhejiang, Hunan and Fujian Provinces. To rescue this critically endangered species, we suggest strengthening field investigations, identifying the exact distribution range and population density of Chinese pangolins and further optimizing the network of nature reserves to improve conservation coverage on the landscape scale and alleviate human interference. Conservation practices that concentrate on the viability assessment of scattered populations could help to improve restoration strategies of the Chinese pangolin.

## Graphical abstract

This study evaluated the effect of anthropogenic and climatic impacts on local extinctions of Chinese pangolins through 300 years of local chronicles and historical environment data in China. Extinction probability increased with population growth (main cause) and climate warming. A quarter of the extant Chinese pangolin population is exposed to notable extinction risk and we need to improve conservation and restoration strategies.

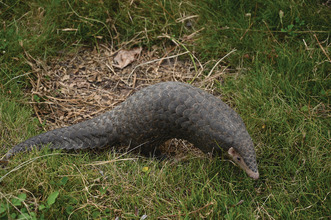

## 1 INTRODUCTION

Accelerated anthropogenic impacts and fluctuating climate change are widely considered to be responsible for the continuous loss of biodiversity (Dirzo et al., 2014; Koch & Barnosky, 2006). Over the past three centuries, many mammals in China have exhibited distinct population declines and shrinking distribution ranges, likely associated with increasing human populations and climate fluctuations (Wan et al., 2019). Habitat loss, population decline or displacement, and even local extinction of wildlife are caused by anthropogenic factors, including over exploitation, agricultural development needs, urbanization, deforestation and human‐introduced diseases (Dirzo et al., 2014; Hill & Hamer, 2004; McKee & Chambers, 2011; Menon et al., 2015; Rosser & Mainka, 2002; Smith et al., 2006; Trombulak & Frissell, 2000; Turvey et al., 2017). Climate change, including warming, cooling and fluctuation, could affect the survival of wildlife regionally, and distribution shifts are the response that would most likely lead to local extinction (Chen et al., 2011; Hei, 2012; IPCC, 2014; Koch & Barnosky, 2006; Li et al., 2015; Pearson & Dawson, 2003). It is widely recognized that human disturbances have the greatest impact on wildlife extinction, but the contribution of climatic factors can also reach up to 54% (Sahajpal & Goyal, 2018; Urban, 2015). In addition, the interaction of climate change and human interference could accelerate wildlife extinction (Wan et al., 2019). Therefore, determining which specific factors have a greater impact will influence conservation decisions.

**FIGURE 1:**
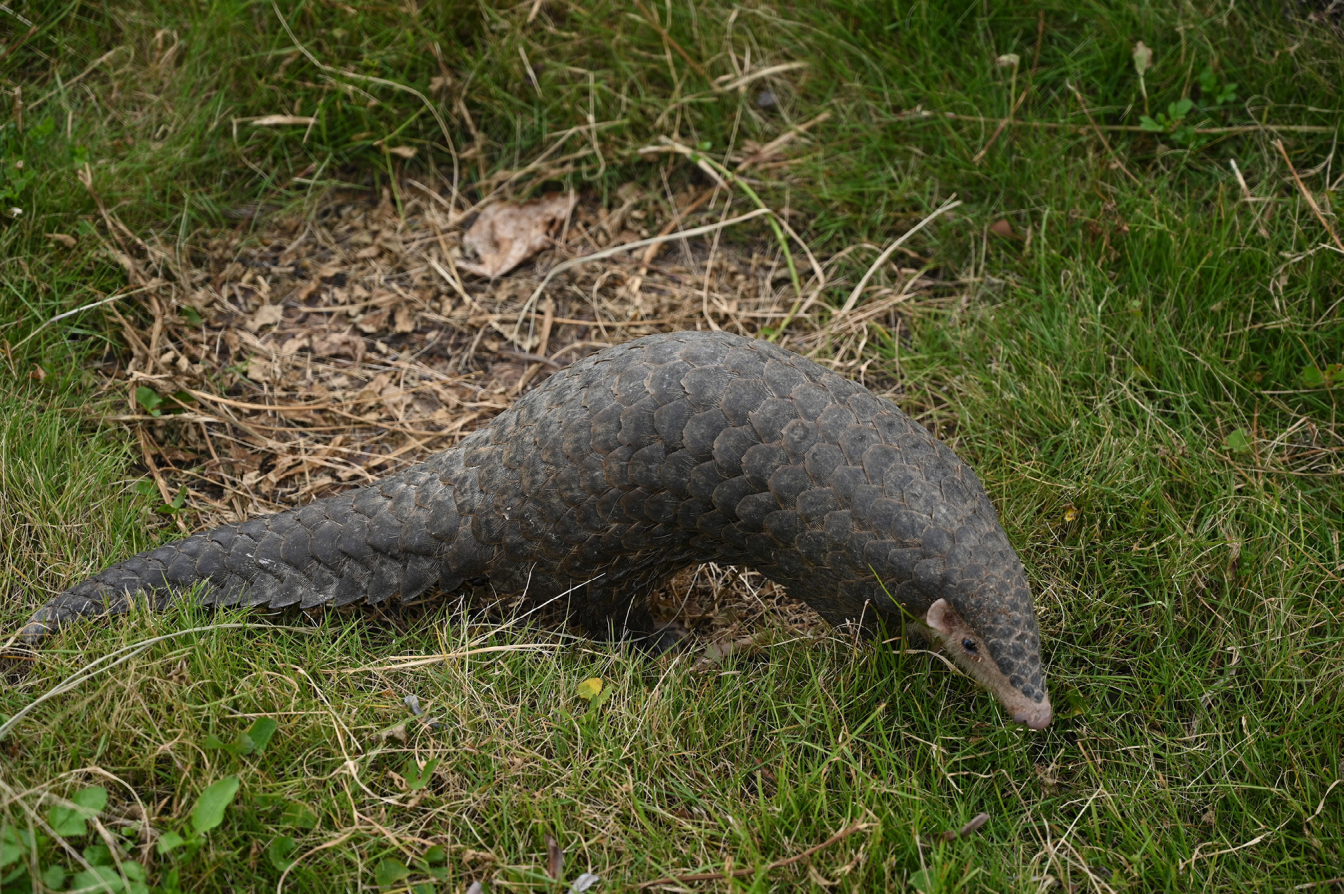
A rescued Chinese pangolin (*Manis pentadactyla*) in the process of rewilding before reintroduction in Guangdong, China (the photo was taken by Yihang Zhang on September 8, 2022).

The Chinese pangolin (*Manis pentadactyla*) is a unique, scale‐covered mammal species that mainly feeds on ants and termites (Figure 1), playing an important role in maintaining the stability of the ecosystem (Liu et al., 2021; Sharma et al., 2020). Up to 2020, China is still one of the largest consumer markets of pangolins and their derivatives in Asia, as pangolin scales are utilized in Traditional Chinese Medicine and their meat is consumed as a luxury food (Sharma et al., 2020). In the past five decades, the distribution range of Chinese pangolins has been rapidly shrinking, and the population has declined by 80–90% in several provinces of China (Challender et al., 2015, 2019). Severe hunting and poaching stress imposed by the local and international pangolin trade across its distribution range has been demonstrated to cause the accelerated decline of Chinese pangolins during this period (D'Cruze et al., 2018; Nash et al., 2016). Despite the enormous human influence, climate change during this period is also very drastic, with the average global surface temperature increasing by approximately 1 °C and extreme weather events occurring more frequently than before (NOAA, 2021). Therefore, we cannot ignore the negative effects of climate change on the loss and alteration of Chinese pangolin habitat (Xian et al., 2022).

The Chinese government has made positive efforts to protect this critically endangered species, such as upgrading all pangolin species from National Level II Protected Animals to Level I, and removing pangolin scales from the *Chinese Pharmacopeia* in 2020 (National Forestry and Grassland Administration, 2020). Identifying the causes of local extinction is an indispensable step in conserving this endangered species (Mateo‐Tomás & Olea, 2010). However, the quantitative relationships between local extinctions of Chinese pangolins and anthropogenic and climatic factors have not yet been evaluated. It is difficult to quantitatively determine which kind of variables are the main factors driving the reduction in Chinese pangolin populations due to the lack of long‐term monitoring data. Local chronicles can provide insight into the roles of human impacts and climate change is causing local extinctions of Chinese pangolins. For more than 3600 years, since the Shang and Zhou dynasties, China has a long history of recording significant political and natural events. Owing to its economic value (mainly for traditional medicine or as a rare gift), unique characteristics and reactions to human activities (curling up when threatened), sightings of Chinese pangolins are likely to be recorded in historical documents. Therefore, local chronicles (from province to district), official and formal records such as *Twenty Four Histories*, and *Comprehensive Mirror for Aid in Government* which record the history of China in annalistic style involves politics, economy, law, military, astronomy, geography and academic culture. In that way, part of historical data describing the Chinese pangolins could be used to track the changes in its distribution range.

In this study, we used local chronicles combined with historical reconstruction of climatic and anthropogenic data to determine the key drivers of local extinctions of Chinese pangolins. Learning from history, our research can have implications for the conservation practices to protect Chinese pangolins in China now and in the future.

## 2 MATERIALS AND METHODS

### 2.1 Establishment of spatio‐temporal GAMs

We fitted local chronicles with quantified anthropogenic stressors and temperature variables using a generalized additive model (GAM) in a 300‐year period. We obtained data regarding the historical distribution of Chinese pangolins from the compendium of *The Distributions and Changes of Rare Wild Animals in China*, in which the occurrence times and locations of the species were recorded from standard histories and local gazetteers, as well as physical remains discovered from 1700 to 2000 AD (Wen, 2009). The approach to the inclusion of historical literature records in the compendium was conservative; records without a clear description and with only a single record in a county were excluded, and only explicit and confirmed records were included (Wen, 2009). We extracted information on Chinese pangolin occurrences (year and location) from the compendium and then reconstructed the longitude and latitude of those locations (Liang, 1980). After the Ming dynasty (1368–1644 AD), the historical administrative divisions of China are recorded more accurately. And therefore, we used the country and prefecture name to directly determine spatial locations. We used ArcGis (version 10.8) to extract center point coordinates of those countries and prefectures from reconstruction maps in each period according to Liang's (1980) book (Qing dynasty, the Republican period and the PRC period). Records for which we could not explicitly determine the coordinates were discarded.

Environmental data are also available at this time. History Database of the Global Environment (HYDE) consists of historical population estimates and land use metrics, in particular, human population count, human population density, cropland coverage and the degree of grazing, which are human factors affecting the survival of wild animals. The HYDE database covers the period from 10,000 BC to 2016 AD, with data from 1700–2016 AD being at a 10‐year temporal resolution and a 0.5 × 0.5 arc degree (approximately 50 × 50 km^2^) spatial resolution (Goldewijk et al., 2017). Weather data reconstructed from the records of δ^18^O in ice cores in the Himalayas and tree rings worldwide exhibit a highly significant correlation with the average temperature and can be used as a metric to assess climate change in the Northern Hemisphere (Shi et al., 2015; Zhao et al., 2014). Benefiting from the unremitting development and updating of historical databases of the global environmental data (anthropic and climatic) and combining local chronicles, we have an opportunity to identify the causes of local extinctions of the Chinese pangolin to inform conservation actions that target the species.

To track the historical extinction events of the Chinese pangolin, we divided China into 4345 square grids (50 km^2^ × 50 km^2^) (Figure S1) and the full study period into ten 30‐year periods (analyses based on 50‐year periods were also conducted, but no significant results were obtained). For each grid, fate was identified as presence and absence for each sampling period. Specifically, if the Chinese pangolin was present in one 30‐year period in a specific grid, we recorded this event as 0 (presence) and if the occurrence of the species was not detected again in this grid, then the next 30‐year period would be recorded as 1 (absence).

Five anthropogenic variables, two climatic variables and the coordinates of extinction events which represent spatial attributes were used to establish GAMs. The anthropogenic factors used in the analysis were as follows: population density, defined as the number of persons per square kilometer per grid cell during each 30‐year period (extracted from HYDE version 3.2.1); cropland, defined as the proportion of cropland coverage in each grid during each period; population counts, defined as the number of inhabitants in each grid; grazing, defined as the proportion of land used for grazing in each grid during each period; and population, defined as the total human population in China during each period (downloaded from https://dataportaal.pbl.nl/downloads/HYDE/HYDE3.2/) (Goldewijk et al., 2017). Oxygen isotopes (δ^18^O) of ice cores in the Tibetan Plateau were used as a proxy for holistic temperature fluctuation in China (downloaded from http://www.tpdc.ac.cn/zh–hans/) (Zhao et al., 2014). Regional temperature (5° × 5° resolution) was represented by the average temperature during the Asian summer (June to August) based on 357 publicly available proxy climate datasets (mainly tree ring sequences) from the World Data Center for Paleoclimatology archives (downloaded from https://www.ncdc.noaa.gov/paleo–search/study/18635) (Shi et al., 2015). From 1700 AD to 2000 AD, the HYDE and climate data had a 10‐year resolution and we therefore used the average during each 30‐year period to represent the entire 30‐year period.

The GAM algorithm was used to model the associations of population density, cropland coverage, population count, grazing, population, temperature and regional temperature with local extinctions of the Chinese pangolin. A Pearson correlation test indicated that the variables of holistic temperature and human population were significantly correlated (*r* = .9788, *p* = .0001), and both those variables exhibited a significant correlation with local extinctions. Comparing the adjusted R^2^ and AIC values in the process of variable screening, we excluded holistic population from the analyses. The GAM analysis was performed using the GAM package in R (version 4.1.2).

### 2.2 Extinction risk assessment

**FIGURE 2:**
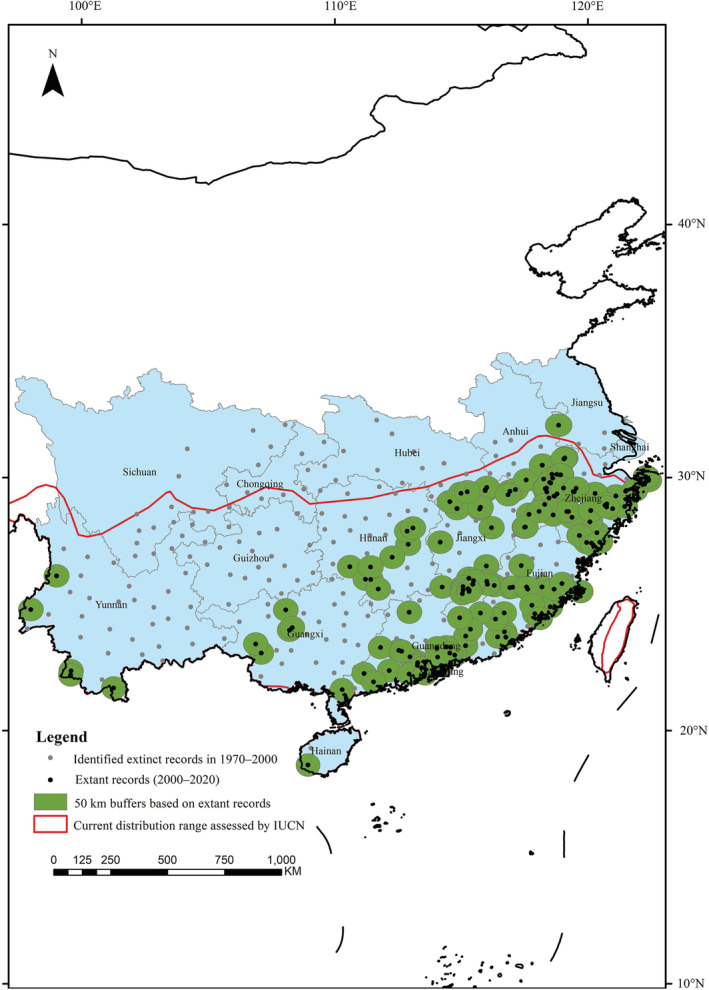
Extant occurrence records, distribution range and identified extinct records of Chinese pangolins across China

We decreased the temporal scale and built an extinction risk model with more sophisticated climatic data using MaxEnt to evaluate the threatened status of extant Chinese pangolin populations (Benito et al., 2009; Rodder et al., 2009). Holistic human population growth and land surface temperature increase in China have been highly correlated in the last three hundred years (Table S1, Pearson correlation test: *r* = .6094, *p* = .0004), which leads to uncertainty regarding which variable plays a stronger role in local extinctions. In total, 604 occurrence records (87% of the historical observations) of Chinese pangolins were documented in 1970–2000 AD across mainland China, and more detailed climatic data are available for this period. We compared those occurrences with the current distribution range of Chinese pangolins assessed by the IUCN expert group to locate extinction records (Challender et al., 2019). We collected 159 rescue and observation records of Chinese pangolins during 2000–2020 AD from the wildlife rescue departments, news reports and GBIF (Global Biodiversity Information Facility) database in China. We set up circular buffer zones (*r* = 50 km) around the extant occurrences of Chinese pangolins to screen for extinction records (Figure 2). Considering the rapid development of wildlife monitoring technology and increased emphasis on biodiversity conservation by the Chinese government, historical records in 1970–2000 AD that fell outside of the current distribution range and buffer zones were considered to be extinction occurrences. Outlying records were tested using the spatial autocorrelation analysis based on Ripley's K function in ArcGis 10.8 (Figure S2). Higher resolution and multidimensional climate data from 1970 to 2000 AD are available from world climate data base (WorldClim, https://www.worldclim.org/). These biological variables relate to various aspects of temperature and precipitation, which in turn affect the geographic distribution of Chinese pangolins and their prey (mainly ants and termites) (Kwon et al., 2014; Li et al., 2018). Therefore, we decreased the time interval and combined the 19 climatic variables from WorldClim, anthropogenic variables from HYDE and identified extinction records of Chinese pangolins to construct a model to assess extinction risk with MaxEnt (Table 1).

**TABLE 1:** Environmental variables and variable‐screening process of extinction risk model. If there existed a high correlation between variables, variable with a higher contribution rate would be selected for model construction.

Variables	Interpretation of variables	Contribution rate (≥1%)	Variables used for model
Bio1	Annual mean temperature		
Bio2	Mean diurnal range (mean of monthly [max temp‐min temp])	√	
Bio3	Isothermality (bio2/bio7) (×100)	√	√
Bio4	Temperature seasonality (standard deviation ×100)	√	
Bio5	Max temperature of warmest month		
Bio6	Min temperature of coldest month	√	
Bio7	Temperature annual range (bio5‐bio6)	√	√
Bio8	Mean temperature of wettest quarter	√	√
Bio9	Mean temperature of driest quarter	√	
Bio10	Mean temperature of warmest quarter		
Bio11	Mean temperature of coldest quarter	√	
Bio12	Annual precipitation	√	
Bio13	Precipitation of wettest month		
Bio14	Precipitation of driest month	√	√
Bio15	Precipitation seasonality (coefficient of variation)		
Bio16	Precipitation of wettest quarter		
Bio17	Precipitation of driest quarter		
Bio18	Precipitation of warmest quarter		
Bio19	Precipitation of coldest quarter		
Cropland	Total cropland area, in km^2^ per grid cell	√	√
Grazing	Total land used for grazing, in km^2^ per grid cell		
Popc	Population counts, in inhabitants/grid cell	√	√
Popd	Population density, in inhabitants/km^2^ per grid cell		
Uopp	Total built‐up area, such as towns, cities, etc, in km^2^ per grid cell	√	

The resolution of the environmental variables was uniform at 5 arc minutes. We set 10 km Euclidean distance to rarefy using SDMtoolbox (Brown, 2014) and ensured that only one extinction occurrence was retained each grid. We input those records and the environmental variables into MaxEnt (version 3.4.1) (Phillips et al., 2006) and ran 25 iterations as pre‐experiment to exclude insignificant variables with 0% contribution and 0 permutation importance value. To avoid multicollinearity of variables, we calculated the Pearson correlation coefficient (*r*) between variables; when *r* > .7, the variable with the lower contribution rate was discarded. Finally, six variables including population counts, cropland, precipitation of driest month (bio14), temperature annual range (bio7), isothermality (bio3) and mean temperature of the wettest quarter (bio8) were used to construct the model (Table 1).

We ran the algorithm 100 times, and the average of the predicted results was output in a logistic format. Maximum training sensitivity plus specificity was used as the threshold value to identify extinction events, and Nature Breaks methods (Jenks, 1967) were used to further assess the levels of extinction probability. The Natural Break is a method of classification that identifies break points by picking the class breaks that best group similar values and maximize the differences between classes. Extant distribution data were derived from occurrence records in GBIF and public information of wildlife management departments. Finally, we extracted the extinction risk of extant populations of Chinese pangolins according to the risk map.

### 2.3 PCA of local extinction events

Through principal component analysis (PCA), we further estimated the variance of related anthropogenic and climatic variables. We identified six variables that contributed to local extinction (contribution rate > 1% in MaxEnt), and we used the sampling method in ArcGis (version 10.8) to extract the information on the associated environmental variables, including population count, bio14, cropland, bio7, bio3, bio8, of the 177 extinction records between1970AD and 2000 AD and to analyze the principal components of local extinction events of Chinese pangolin. The PCA was performed by FactoMineR and factoextra packages in R (version 4.1.2).

## 3 RESULTS

### 3.1 Effects of anthropic and climatic factors

**FIGURE 3:**
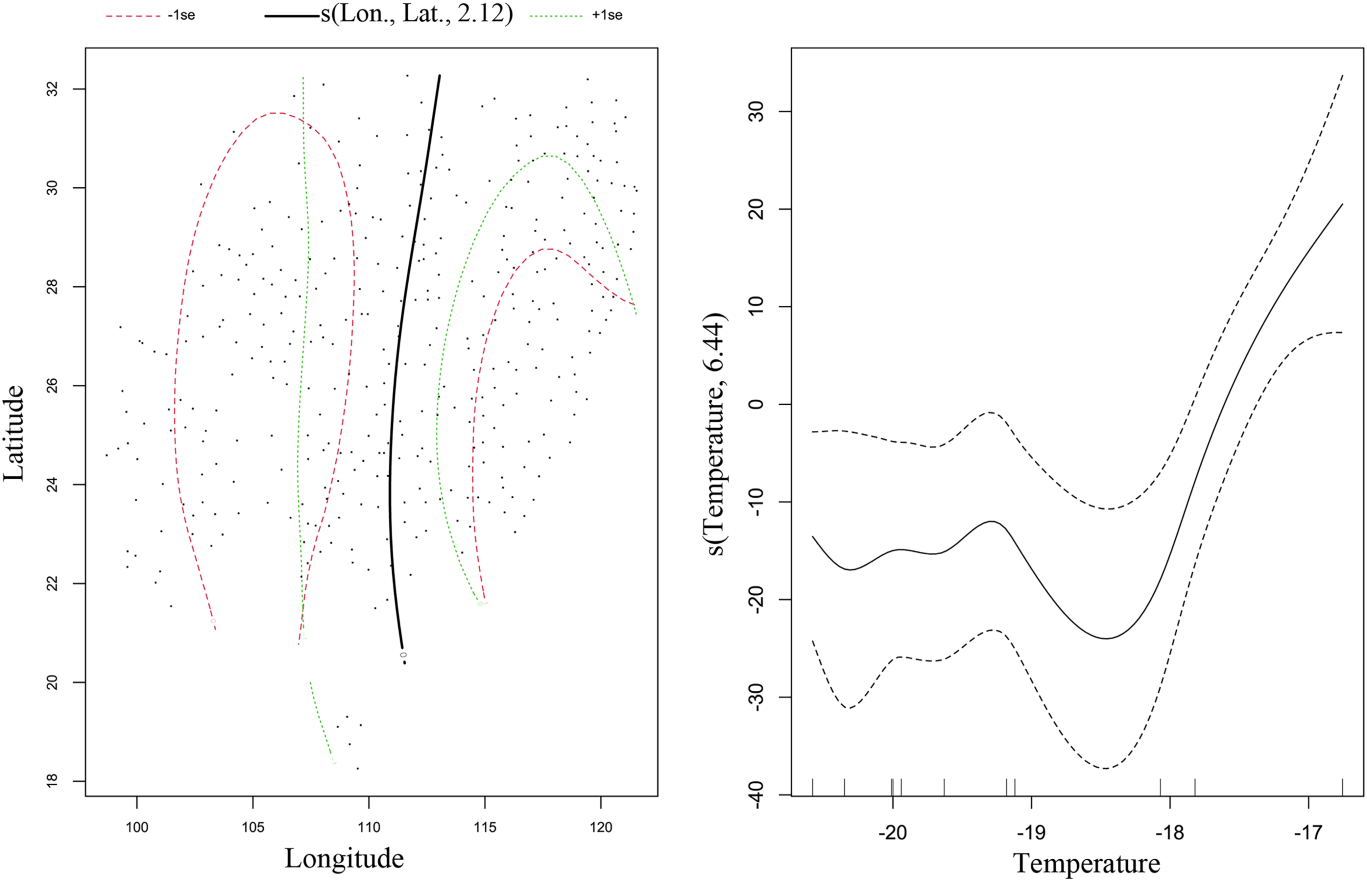
Relationship between local extinctions and temperature, geographical distribution from 1700 AD to 2000 AD. Local extinctions are dichotomous events (0,1), and temperature is inferred from oxygen isotopes.

After data verification, we detected 361 local extinction events of Chinese pangolins across China. Through spatio‐temporal GAM analysis, we found that temperature was significantly positively correlated with the local extinctions of Chinese pangolin between 1970 AD and 2000 AD across mainland China (Table 2). The extinction probability of the Chinese pangolin increased with rising temperature (Figure 3). In addition, Chinese pangolins were more likely to go extinct at higher longitudes and latitudes in mainland China (Figure 3). However, the effects of population density, cropland, grazing and regional temperature on local extinctions of Chinese pangolin were not detected through this method (Table 2). The opinion that climate change and human interference affected the survival and geographical distribution of Chinese pangolins was supported. However, we could still not determine which variable played a pivotal role in local extinctions of Chinese pangolins according to the regression models.

**TABLE 2:** Correlation coefficient and significance testing of the established GAMs. N = 722

Variables	Coefficients	Adjusted R^2^	Deviance explained
Temperature	6.44***	0.928	91%
Lon., Lat.	2.11*		
Popd	5.02		
Popc	1.037		
Corpland	1.638		
Grazing	1.037		
RegionalTemp.	1.074		

*Note*: *p < .05, *** *p* < .001.

Abbreviations: Lat., latitude; Lon., longitude.

### 3.2 Chinese pangolin extinction‐risk model

**FIGURE 4:**
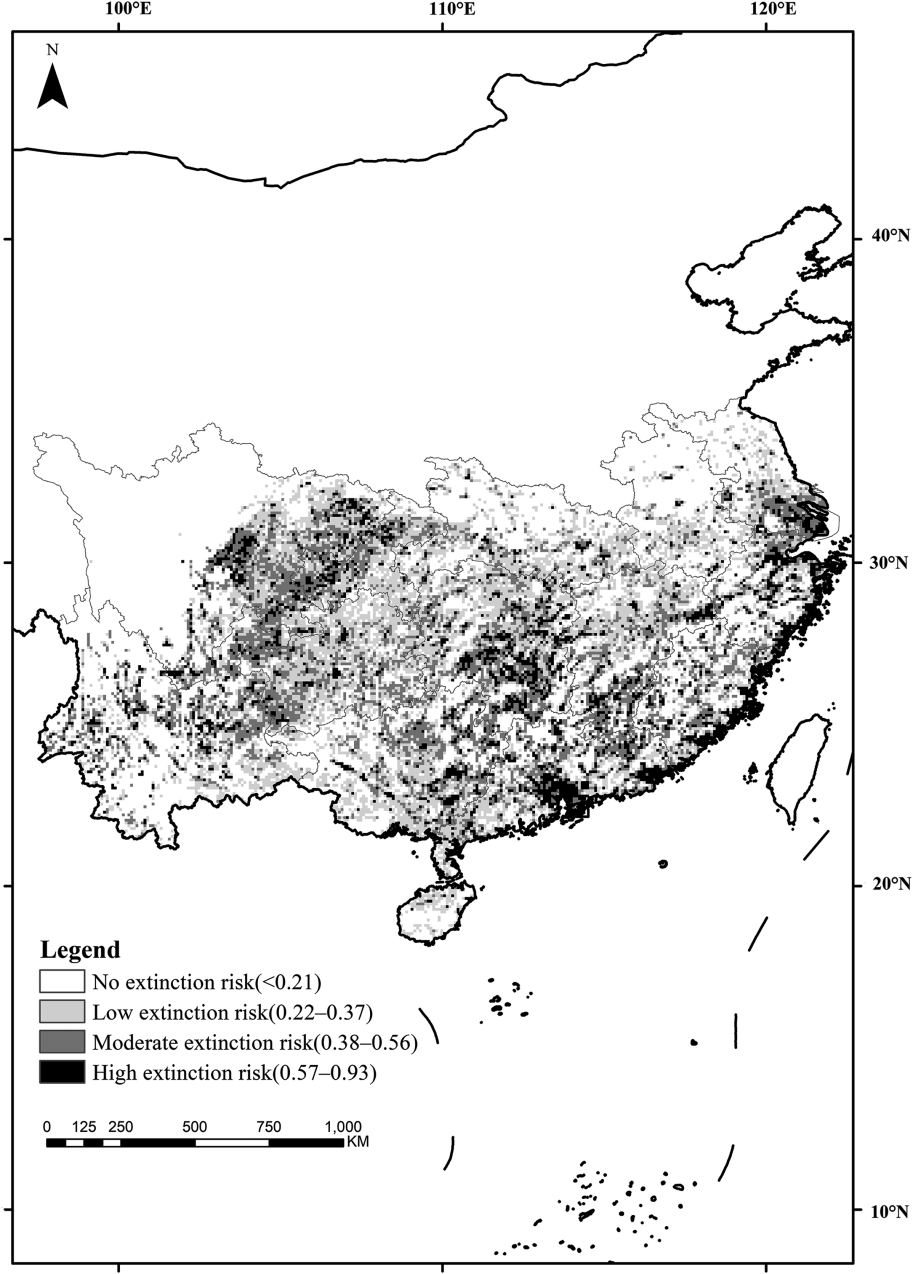
Extinction‐risk assessment of Chinese pangolins across China predicted by MaxEnt

We found that 35 Chinese pangolin occurrences were outside the current distribution range and 142 occurrences were outside of the buffer zones of extant distribution occurrences. In total, 177 dispersed records were used to model the extinction risk of the Chinese pangolin across China with MaxEnt between 1970 AD and 2000 AD (Figure 2). The average test AUC of 100 replicate runs was 0.94, and the standard deviation was .01. The prediction results had a satisfactory reference value. The population count contributed 50.90% towards the final model, bio14 contributed 29.40%, and bio7 contributed 11.30%, with the other variables contributing <5% each. The extinction risk ranged from 0 to 0.93 (Figure 4). The marginal response curve of a single variable indicated that as the population count and precipitation in driest month increased, the extinction risk was elevated exponentially. When the population count reached approximately 7000–8000 persons in each grid (supersaturated status), the extinction risk remained almost unchanged. The extinction risk first increased and then decreased with the annual range of temperature. The extinction risk model indicated that anthropogenic variables could be the principal causes of local extinctions of Chinese pangolins, followed by climatic variables.

### 3.3 Variation in environmental variables

**FIGURE 5:**
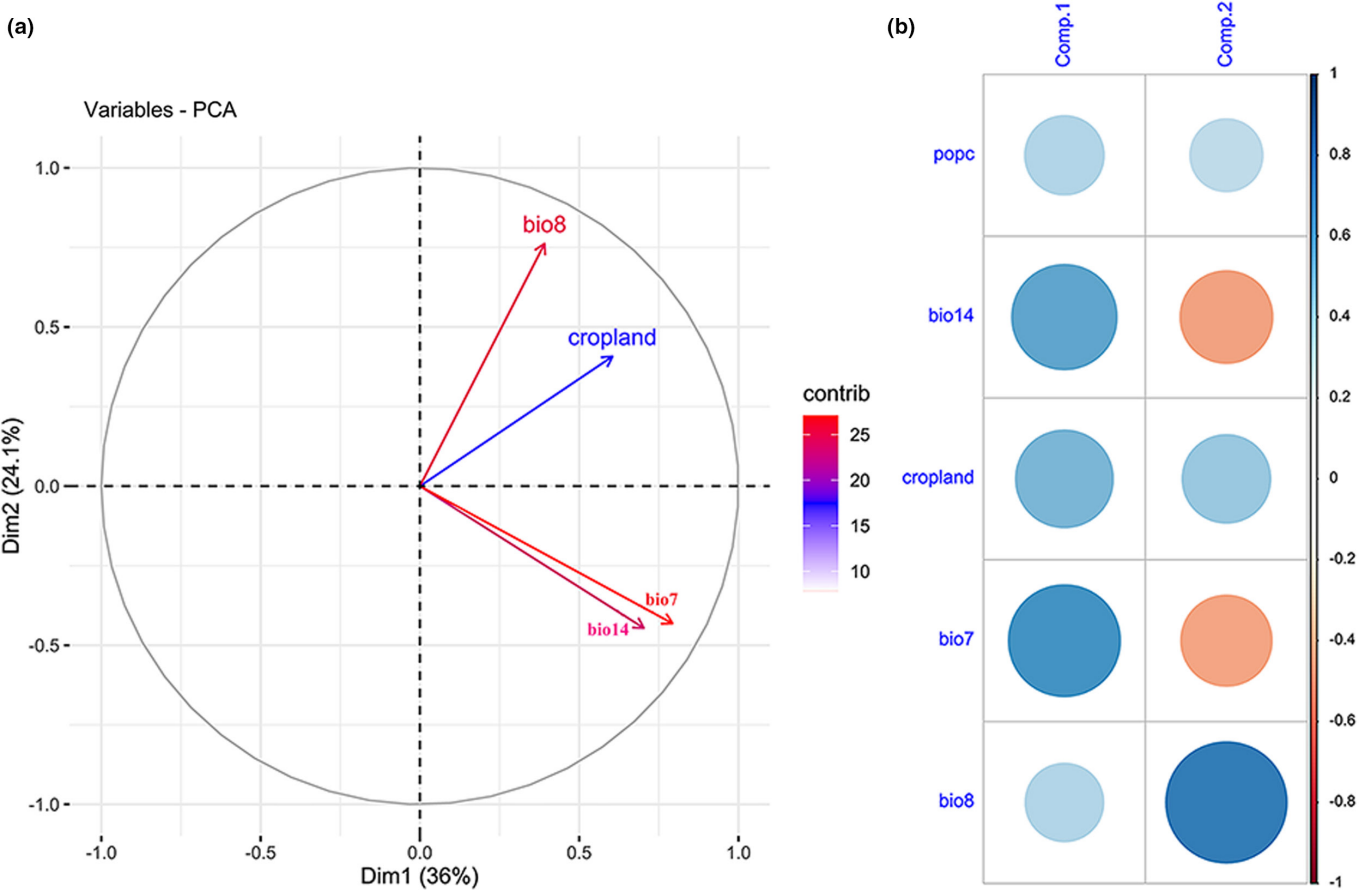
Variable contribution rate of the first two principal components (a) and representation of principal components to variables (b)

Through PCA, we analyzed the environmental information of the 177 extinction occurrences of Chinese pangolins outside of the distribution range between1970AD and 2000 AD across China. After dimension reduction, Comp. 1 represented the temperature annual range (bio7, positive correlation) and precipitation of the driest month (bio14, positive correlation). Comp. 2 represented the mean temperature of the wettest quarter (bio8, negative correlation) and the proportion of cropland (positive correlation). The first two principal components accounted for 60.16% of the variance (Figure 5). The PCA results indicated that climatic factors had a greater degree of variation than anthropic variables in extinction events.

### 3.4 Extinction–risk assessment of extant populations

**FIGURE 6:**
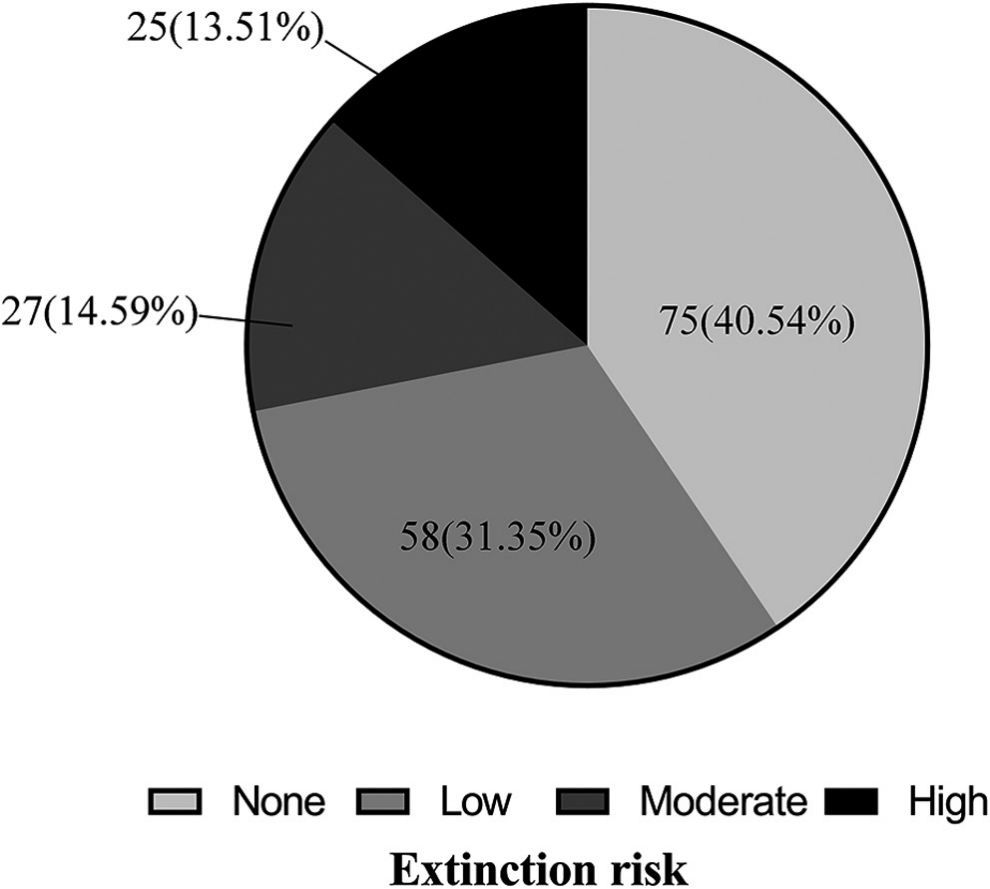
Extant Chinese pangolin populations facing extinction risk

Through the sampling method in ArcGis, we extracted the extinction probability of extant distribution sites according to the extinction‐risk map of Chinese pangolins. The results showed that 40.54% of extant distribution sites were confronted with no extinction risk (extinction probability < 0.21), 31.35% were at low extinction risk (0.22–0.37), 14.59% were at moderate extinction risk (0.38–0.56) and 13.51% were at high extinction risk (0.57–0.93) (Figure 6). In total, more than a quarter of extant populations were at notable (moderate and high) extinction risk. Those sites with moderate and high extinction risk were predominantly distributed in southeast China, including Guangdong, Jiangxi, Zhejiang, Hunan, Fujian, and Jiangsu Provinces. Of the 52 sites that were at moderate and high risk of extinction, 16 sites were confronted with moderate and high extinction risk spread over Guangdong Province, 8 sites were in Jiangxi and Zhejiang Provinces, and 4 sites were in Hunan and Fujian Province.

## 4 DISCUSSION

Combining multiple perspectives, scales and methods, local chronicles revealed that anthropic and climatic variables were significantly associated with local extinctions of Chinese pangolins in China. In summary, the main factor was intensive human interference, while global warming could accelerate the extinction process. Hunting, farming and grazing conducted by humans caused population declines and the extinction of wildlife. The anthropic pressure to which Chinese pangolins were exposed might have exceeded their tolerance threshold. Through the interaction of human and climate disturbances, more drastic climate change in recent years has accelerated the extinction rate of Chinese pangolins (Li et al., 2018). Our results imply that human disturbance and climate change co‐determined the current distribution of Chinese pangolins. The population and distribution range of the Chinese pangolin will continue to shrink with highly intensive human activities and drastic climate change. Local chronicles serve as long‐term monitoring data and provide important insights revealing the association of anthropic and climatic variables with the local extinctions of the Chinese pangolin. The Chinese pangolin was once widely distributed in China (Allen & Coolidge, 1940), and is now only distributed in 11 provinces, including Taiwan, Zhejiang, Guangdong, Fujian, Jiangxi, Anhui, Yunnan, Hongkong, Chongqing, Hainan and Guangxi provinces (Kong et al., 2021). From the 1960s, the population of the Chinese pangolin decreased by 88.88–94.12% and disappeared from more than half of its distribution range in southern China (Wu et al., 2004). Historical data have helped identify the driving forces of local extinctions in the long term and have contributed to understanding the current distribution pattern of Chinese pangolins. However, due to the limitations of historical environmental data, hunting and poaching pressures could not be accurately assessed because measurements of population density and counts do not exhibit a correlation with them in highly urbanized areas (Nash et al., 2016). This could be the reason why GAMs were unable to detect the significant influence of human population density and count.

Along with human population growth, trade and consumer demand have become major threats to the survival of Chinese pangolins and can be represented by the total human population count (Challender et al., 2015). Pangolin scales are thought to cure evil sores, malaria, and mastopathy according to traditional Chinese medicine (e.g. *Compendium of Materia Medica*) and pangolin meat is considered a luxury food item (Challender et al., 2015). China is one of the largest consumer markets in Southeast Asia, and a growing population has led to increasing demands for pangolin products. In addition, human population growth exacerbates hunting, poaching, and land utilization, which directly leads to the decline of pangolin populations, habitat loss and fragmentation, followed by local extinctions (Turvey et al., 2017).

Though the total human population and the average temperature of China both present increasing trends, the differences in temperature are much more fluctuant (Table S1). Based on the results of the principal component analysis, the variation of climate data was much greater than that of anthropogenic data, implying that the Chinese pangolin could be more easily affected by climate change in the future. The Chinese pangolin is a homeotherm and accelerated global warming and temperature fluctuations may affect them negatively. First, the density of Chinese pangolins may change at given locations, and the ranges of species may shift either poleward or up in elevation as species move to occupy areas with climates within their metabolic temperature tolerances (Subba et al., 2018). Second, because many natural history traits of species are triggered by temperature‐related cues, changes could occur in the timing of events (phenology), such as migration and breeding (Both & Marvelde, 2007). The synergism of rapid temperature rises and human stressors, in particular habitat destruction, could easily disrupt the connectedness among species and lead to a reformulation of species communities, reflecting differential changes in species, and to numerous extirpations and possibly extinctions (Root et al., 2003). In addition, global warming increases the probability of extreme weather and wildlife diseases (Harvell et al., 2009; Vaughan, 2019). East Asia is subject to increase warm and dry extremes, and southeast Asia experiences a higher probability of extreme rainfall in spring (Lee et al., 2012; Thirumalai et al., 2017). The risk of contracting diseases (especially vector‐borne diseases) both in humans and animals increases as a result of global warming (Massad et al., 2011). Species extinctions may be due to changes in habitats or the transport of livestock which facilitates the movement of viruses and arthropods (especially ticks) from one place to another (Black et al., 2008; Dhama et al., 2013).

Their scattered distribution implies that the conservation practices of Chinese pangolins must depend on the efforts of local governments. Chinese pangolin populations with a high risk of extinction are spread over more than six provinces in China, and 30.77% of high‐risk populations are distributed in Guangdong, which is one of the most developed provinces in China. Therefore, the challenge is how to coordinate wildlife conservation and local economic development in Guangdong. Given this, we suggest strengthening population field investigations and accurately identifying the distribution range of Chinese pangolins. Further efforts to optimize the network of nature reserves to improve the conservation coverage of Chinese pangolins from the perspective of territory are required because the species had not received enough conservation resources in the past. An ex‐situ conservation strategy is another workable solution to overcome conflicts between local economic development and small populations of Chinese pangolins (Vitt & Havens, 2004).

The Chinese government has been strengthening its conservation policy for the Chinese pangolin. All illegal wildlife trade has been strictly banned to eliminate the excessive consumption of wildlife and ensure ecological security (National People's Congress, 2020). In addition, the Chinese government strengthened the management of medicinal animal products, and pangolin scales were removed from the Chinese pharmacopeia in 2020 (National Medical Products Administration, 2020). Moreover, the Chinese government upgraded the designation of the Chinese pangolin to first‐class national protected animals in the same year (National Forestry and Grassland Administration, 2020), indicating that this species and its habitat would receive stricter protection after the prohibition of the wildlife trade.

Future conservation practices need to focus more attention on assessing the long‐term viability of small populations and the subsequent population restoration. Excessive human exploitation and utilization of land resources leads to habitat fragmentation and suitable habitat patches and even national nature reserves with defined protection objectives usually present as “isolated islands” in a world dominated by human activities. Small populations are less stable and more susceptible to inbreeding depression and outside interference (Seth et al., 2021). Chinese pangolins are widely distributed, and it is feasible to establish ecological corridors between habitat patches that are in close proximity. However, species recovery among long‐distance and isolated patches requires appropriate human intervention to save local populations. After a thorough assessment of habitat and ecological risk, artificial breeding, rewilding and reintroduction are reasonable methods to guard against the extinction of small populations (Kuehler et al., 2000). In addition, based on an empirical analysis, the distribution mode of extant Chinese pangolins did not show a typical avoidance strategy for human settlements (Wang et al., 2020; Zhang et al., 2021). Residential and rural areas that overlap with pangolin populations should be targeted for the efforts to improve awareness of the benefits of wildlife conservation (Zhang et al., 2021).

## AUTHOR CONTRIBUTIONS

**Haiyang Gao:** Formal analysis (lead); methodology (lead); software (lead); validation (lead); visualization (lead); writing – original draft (lead). **Hongliang Dou:** Data curation (equal); funding acquisition (equal); methodology (equal); visualization (equal). **Shichao Wei:** Data curation (equal); formal analysis (equal); methodology (equal); visualization (equal). **Song Sun:** Data curation (equal); formal analysis (equal); software (equal); validation (equal); visualization (equal). **Yulin Zhang:** Formal analysis (equal); methodology (equal); software (equal); validation (equal); visualization (equal). **Yan Hua:** Data curation (equal); funding acquisition (lead); methodology (equal); project administration (lead); supervision (lead); writing – review and editing (lead).

## CONFLICT OF INTEREST

The authors declare no conflicts of interest.

### OPEN RESEARCH BADGES

This article has earned an Open Data badge for making publicly available the digitally‐shareable data necessary to reproduce the reported results. The data is available at https://doi.org/10.5061/dryad.0gb5mkm4d.

## Supporting information


Appendix S1

## Data Availability

Data used in this study are available at the Dryad Digital Repository: https://doi.org/10.5061/dryad.0gb5mkm4d.
