# Supplementary material for: Local chronicles reveal the effect of anthropogenic and climatic impacts on local extinctions of Chinese pangolins (Manis pentadactyla) in mainland China
Source: Ecol Evol. 2022 Oct 5;12(10):e9388. doi: 10.1002/ece3.9388 (PMC9534744; doi:10.1002/ece3.9388)
Supplement: Supplementary file 1 — Appendix S1 [file ECE3-12-e9388-s001.docx]

**Appendix S**

Appendix S includes one table (Table S1) and two figures from figure S1 to S2, which are cited in the main text. Table S1 shows that there exists a high correlation between climate warming and population increase in China from 1700AD to 2000AD. Figure S1 exhibits the fishnet division of mainland China at a 50 km resolution and historical records of Chinese pangolins at this period. Figure S2 is spatial autocorrelation test of distribution records using Ripleys’ K function in 1970–2000AD. Test result suggests extinction records we identified are dispersed and can be analyzed in MaxEnt.

**Table S1.** Temperature increase inferred from oxygen isotope and human population growth in China. Pearson correlation test: *r*=0.6094, *p*<0.01.

| **Year** | **Temperature** | **Total human population** |
| --- | --- | --- |
| 1700 | -19.42 | 99429248 |
| 1710 | -19.93 | 104046300 |
| 1720 | -20.68 | 119498000 |
| 1730 | -19.77 | 127053450 |
| 1740 | -21.99 | 142975500 |
| 1750 | -19.29 | 178327220 |
| 1760 | -19.63 | 197045540 |
| 1770 | -21.08 | 220129390 |
| 1780 | -21.04 | 275682140 |
| 1790 | -21.52 | 298722910 |
| 1800 | -18.67 | 292008260 |
| 1810 | -19.63 | 341920510 |
| 1820 | -20.67 | 352710590 |
| 1830 | -19.35 | 388372160 |
| 1840 | -19.97 | 410874820 |
| 1850 | -19.72 | 429199200 |
| 1860 | -18.98 | 421916160 |
| 1870 | -20.2 | 358028350 |
| 1880 | -18.65 | 362239840 |
| 1890 | -18.65 | 373600670 |
| 1900 | -20.24 | 398031620 |
| 1910 | -18.58 | 420924190 |
| 1920 | -19.78 | 469681440 |
| 1930 | -19.01 | 486599330 |
| 1940 | -18.41 | 518286590 |
| 1950 | -17.14 | 534354750 |
| 1960 | -17.91 | 631503810 |
| 1970 | -17.97 | 810440450 |
| 1980 | -17.67 | 978000190 |
| 1990 | -18.58 | 1139202900 |


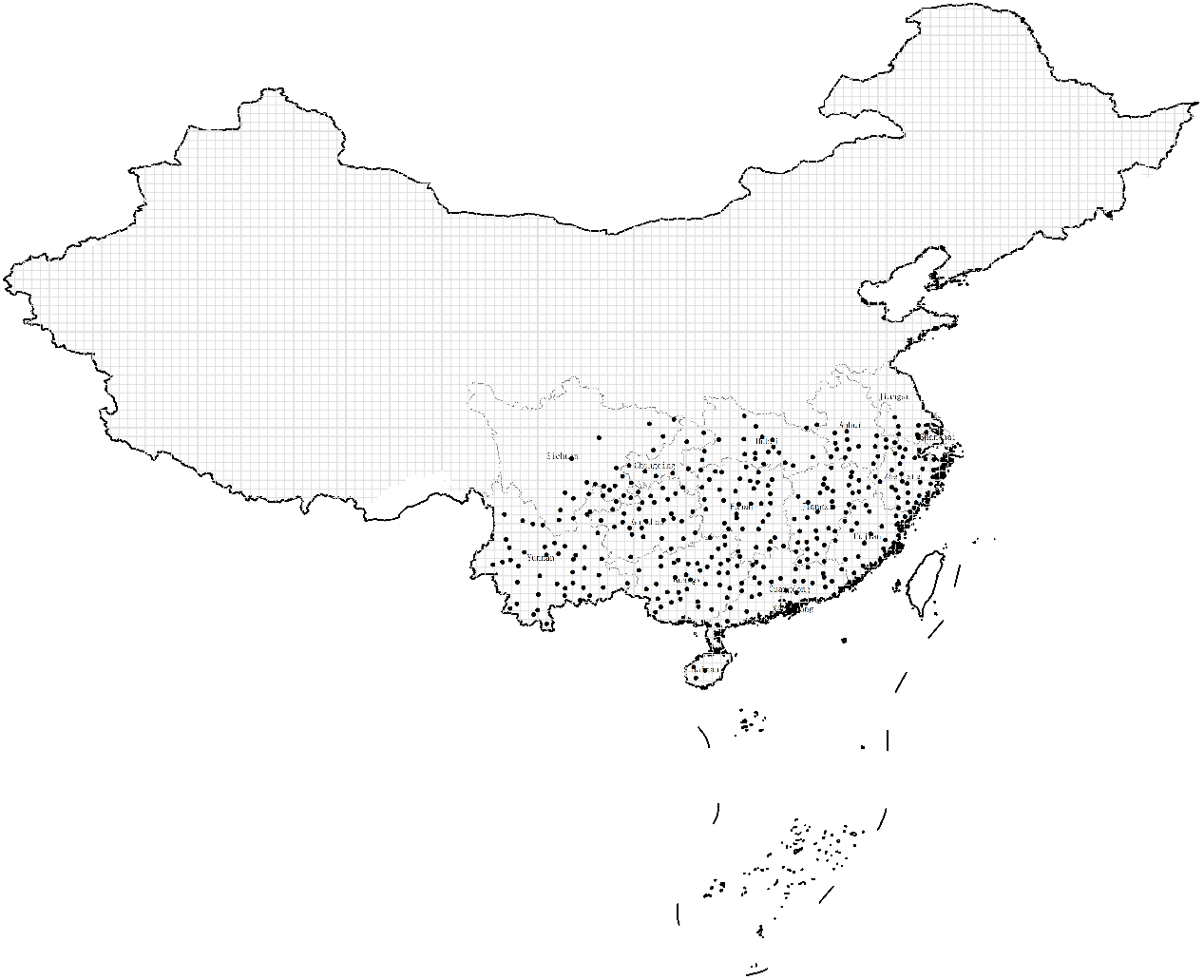


**Figure S1.** Fishnet of mainland China at a 50 km resolution and historical distribution of Chinese pangolins between 1700AD and 2000AD.

**
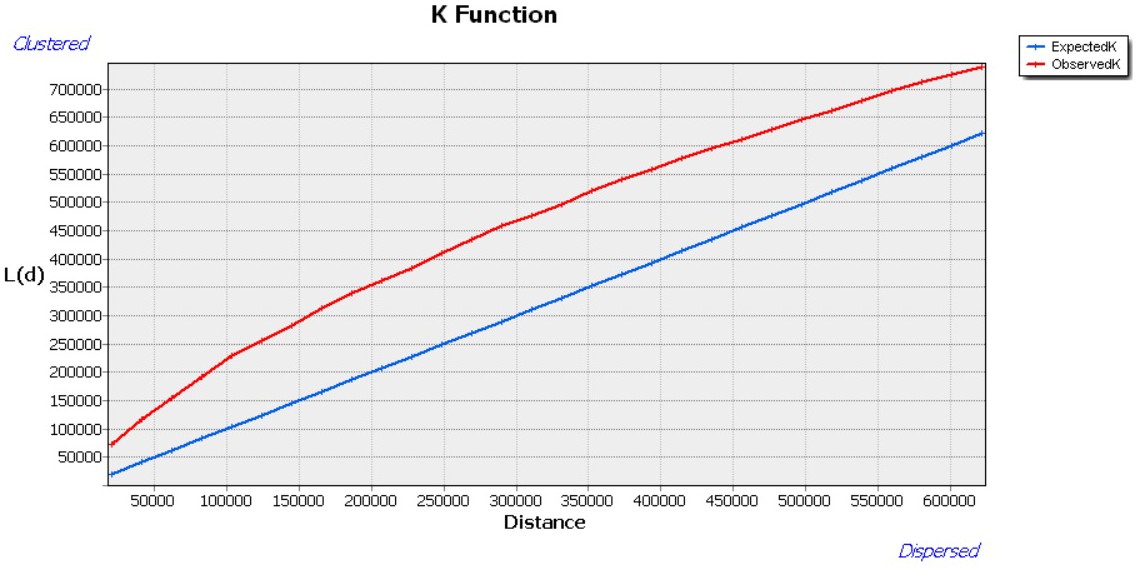
**

**Figure S2.** Auto-correlation test of identified extinction records through Ripleys’ K function.
